# Comparison of the effects of renal denervation at early or advanced stages of hypertension on cardiac, renal, and adipose tissue pathology in Dahl salt-sensitive rats

**DOI:** 10.1038/s41440-024-01605-x

**Published:** 2024-02-15

**Authors:** Kohzo Nagata, Kaito Tagami, Touko Okuzawa, Misaki Hayakawa, Akane Nomura, Tomo Nishimura, Katsuhide Ikeda, Kento Kitada, Shuhei Kobuchi, Yoshihide Fujisawa, Akira Nishiyama, Toyoaki Murohara

**Affiliations:** 1 Pathophysiology Sciences, Department of Integrated Health Sciences, Nagoya University Graduate School of Medicine, Nagoya, Japan; 2 Department of Medical Technology, Nagoya University School of Health Sciences, Nagoya, Japan; 3 Department of Pharmacology, Faculty of Medicine, Kagawa University, Kagawa, Japan; 4 Division of Pharmacology, School of Pharmacy, Department of Pharmacy, Hyogo Medical University, Kobe, Japan; 5 Department of Cardiology, Nagoya University Graduate School of Medicine, Nagoya, Japan

**Keywords:** Adipose inflammation, Cardiac remodeling, Renal denervation, Renal injury, Salt-sensitive hypertension

## Abstract

Renal denervation (RDN) has emerged as a novel therapy for drug-resistant hypertension. We here examined the effects of RDN at early versus advanced stages of hypertension on blood pressure and organ pathology in rats with salt-sensitive hypertension. Dahl salt-sensitive (DahlS) rats fed an 8% NaCl diet from 6 weeks of age were subjected to RDN (surgical ablation and application of 10% phenol in ethanol) or sham surgery at 7 (early stage) or 9 (advanced stage) weeks and were studied at 12 weeks. RDN at early or advanced stages resulted in a moderate lowering of blood pressure. Although RDN at neither stage affected left ventricular (LV) and cardiomyocyte hypertrophy, it ameliorated LV diastolic dysfunction, fibrosis, and inflammation at both stages. Intervention at both stages also attenuated renal injury as well as downregulated the expression of angiotensinogen and angiotensin-converting enzyme (ACE) genes and angiotensin II type 1 receptor protein in the kidney. Furthermore, RDN at both stages inhibited proinflammatory gene expression in adipose tissue. The early intervention reduced both visceral fat mass and adipocyte size in association with downregulation of angiotensinogen and ACE gene expression. In contrast, the late intervention increased fat mass without affecting adipocyte size as well as attenuated angiotensinogen and ACE gene expression. Our results thus indicate that RDN at early or late stages after salt loading moderately alleviated hypertension and substantially ameliorated cardiac and renal injury and adipose tissue inflammation in DahlS rats. They also suggest that cross talk among the kidney, cardiovascular system, and adipose tissue may contribute to salt-sensitive hypertension.

**Figure:**
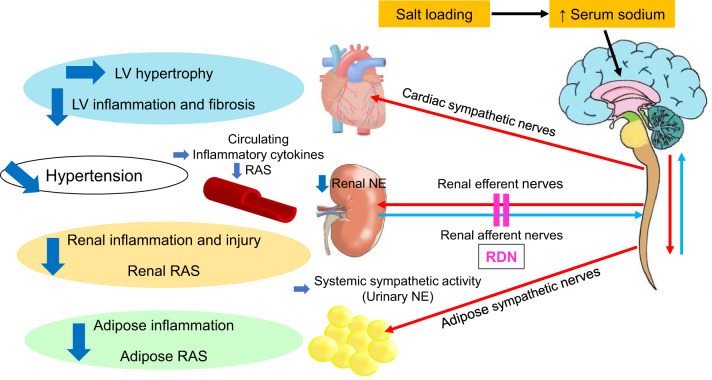
Supposed mechanism for the beneficial effects of RDN on hypertension and target organ damage in DahlS rats. RDN at early or late stages after salt loading moderately alleviated hypertension and substantially ameliorated renal injury in DahlS rats. Cross talk among the kidney, cardiovascular system, and adipose tissue possibly mediated by circulating RAS may contribute to salt-sensitive hypertension. LV; left ventricular, NE; norepinephrine, RAS; renin-angiotensin system, RDN; renal denervation.

Supposed mechanism for the beneficial effects of RDN on hypertension and target organ damage in DahlS rats. RDN at early or late stages after salt loading moderately alleviated hypertension and substantially ameliorated renal injury in DahlS rats. Cross talk among the kidney, cardiovascular system, and adipose tissue possibly mediated by circulating RAS may contribute to salt-sensitive hypertension. LV; left ventricular, NE; norepinephrine, RAS; renin-angiotensin system, RDN; renal denervation.

## Abstract (web summary)

Supposed mechanism for the beneficial effects of RDN on hypertension and target organ damage in DahlS rats. RDN at early or late stages after salt loading moderately alleviated hypertension and substantially ameliorated renal injury in DahlS rats. Cross talk among the kidney, cardiovascular system, and adipose tissue possibly mediated by circulating RAS may contribute to salt-sensitive hypertension. LV; left ventricular, NE; norepinephrine, RAS; renin-angiotensin system, RDN; renal denervation.

## Introduction

Hypertension is a major cause of premature death worldwide, but its underlying mechanisms remain poorly understood, as is highlighted by the large number of individuals with uncontrolled high blood pressure. Renal sympathetic nerves have been identified as a major contributor to the pathophysiology of hypertension in both experimental models and humans [1]. Most individuals with essential hypertension have an increased efferent sympathetic drive to the kidneys, as is evidenced by increased renal norepinephrine spillover. Hypertension is also characterized by an increased rate of sympathetic nerve firing, which is possibly modulated by afferent signaling from renal sensory nerves [2]. Catheter-based renal denervation (RDN) has become available in clinical trials as a possible treatment option for drug-resistant hypertension [3].

Sustained overactivity of the sympathetic nervous system, including renal nerves, is thus thought to represent a pathophysiological mechanism of treatment-resistant hypertension. Excretion of norepinephrine and the pressor response during elevation of the sodium concentration in cerebrospinal fluid were found to be similar in prehypertensive Dahl salt-sensitive (DahlS) rats and their normotensive controls (Dahl salt-resistant rats), suggesting that sympathetic nerve activity does not differ between the two strains [4]. DahlS rats become hypertensive when fed a high-salt diet, with such hypertension being accompanied by increased sympathetic activity as well as increased renal and splanchnic vascular resistance [5]. Also, in obese DahlS rats, salt-induced sympathetic overactivity increased lipolysis and inflammation in adipose tissue as well as exacerbated insulin resistance and systemic inflammation, thereby contributing to enhanced hypertension [6]. Although RDN has consistently been found to have no effect on the development of hypertension in DahlS rats [7], it has been shown to attenuate hypertension after high salt intake in this model [5].

Whereas first-generation clinical trials of RDN demonstrated its safety and efficacy for lowering blood pressure in individuals with drug-resistant hypertension [8, 9], results from the more robustly designed Symplicity HTN-3 trial raised questions about such efficacy [10]. Subsequent favorable results from the SPYRAL HTN Global Clinical Trial Program rekindled interest in the use of RDN for the treatment of hypertension [11]. Although the Symplicity HTN-Japan trial was underpowered for analysis of the primary endpoint, promising results were obtained with a Japanese hypertensive population characterized primarily by high salt intake, high salt sensitivity, and a greater risk for stroke than their white counterparts in earlier studies [12]. These findings have suggested that RDN might be most effective for the treatment of hypertension associated with sympathetic overactivation, such as salt-sensitive hypertension [13]. We have now investigated the effects of RDN in early versus advanced stages of hypertension on blood pressure as well as on cardiac, renal, and adipose tissue pathology in rats with salt-sensitive hypertension.

## Methods

### Animals and experimental protocols

All animal experiments were approved by the Animal Experiment Committee of Nagoya University Graduate School of Medicine (Daiko district, approval nos. 1902, 20018, D210014-001, and D220009-002). Five-week-old male inbred DahlS rats were obtained from Japan SLC (Hamamatsu, Japan) and were handled in accordance with the guidelines of Nagoya University Graduate School of Medicine as well as with the Guide for the Care and Use of Laboratory Animals (NIH publication no. 85–23, revised 2011). They were fed a low-salt diet containing 0.3% NaCl until 6 weeks of age and a high-salt diet containing 8% NaCl thereafter. They were allowed free access to the diet and tap water throughout the experimental period. The rats were randomly assigned to four groups at 6 weeks of age and were subjected to sham surgery or RDN at 7 weeks [sham (7w) group (*n* = 11) and RDN (7w) group (*n* = 11), respectively] or 9 weeks [sham (9w) group (*n* = 11) and RDN (9w) group (*n* = 12), respectively]. RDN was performed by surgical ablation and by painting with a solution of 10% phenol in absolute ethanol [14, 15]. Body weight and food intake were measured weekly. At 12 weeks of age, rats were placed in metabolic cages for the collection of 24-h urine specimens and then subjected to echocardiography. The animals were then killed, and the heart, kidneys, and visceral (retroperitoneal) fat tissue were removed and weighed. Left ventricular (LV) tissue was also separated from the heart for analysis. The kidneys were cut vertically in the center to prepare tissue specimens for histological examination, while the remaining tissues were used for quantitative RT-PCR analysis, immunoblot analysis, and measurement of renal norepinephrine content.

### Hemodynamic and echocardiographic analyses

Systolic blood pressure (SBP) and heart rate were measured weekly before RDN and twice a week after RDN in conscious animals by tail-cuff plethysmography (BP-98A; Softron, Tokyo, Japan) [16]. At 12 weeks of age, rats were anesthetized by i.p. injection of ketamine (50 mg/kg) and xylazine (10 mg/kg) for echocardiographic analysis, also as previously described [17].

### Measurement of renal norepinephrine content

RDN was assumed to have been achieved by confirmation that renal norepinephrine content at 12 weeks of age was <10% of the mean value in the corresponding sham-operated group [15]. Renal norepinephrine content was measured by high-performance liquid chromatography as described previously [7, 18].

### Other methods

Histological and immunohistochemical analysis, biochemical analysis, reverse transcription (RT) and real-time polymerase chain reaction (PCR) analysis, and immunoblot analysis are described in Supplementary Information.

### Statistical analysis

Data are presented as means ± SEM. Differences between groups of rats at 12 weeks of age were assessed by the unpaired Student’s *t* test. Time courses of body weight, food intake, SBP, and heart rate were compared by two-way repeated-measures analysis of variance (ANOVA). A *P* value of <0.05 was considered statistically significant.

## Results

### Physiological parameters and renal norepinephrine content

**Fig. 1:**
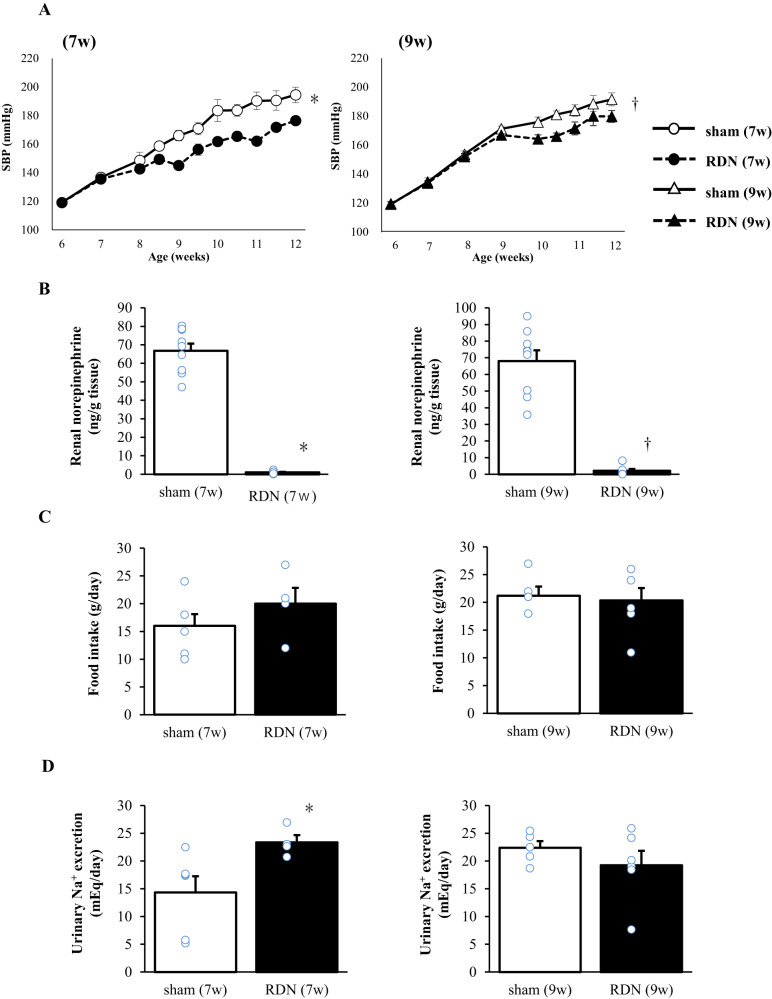
Time course of SBP **A** as well as renal norepinephrine content **B**, food intake **C**, and urinary Na^+^ excretion **D** at 12 weeks of age for rats subjected to sham surgery or RDN at 7 or 9 weeks of age. Data are means ± SEM for surviving animals [SBP, *n* = 11, 11, 11, and 12 at 6 weeks and *n* = 8, 11, 9, and 10 at 12 weeks; renal norepinephrine content at 12 weeks, *n* = 9, 9, 9, and 10; food intake at 12 weeks, *n* = 6, 4, 5, and 6; and urinary Na^+^ excretion at 12 weeks, *n* = 6, 4, 5, and 6 for sham (7w), RDN (7w), sham (9w), and RDN (9w) rats, respectively]. *P < 0.05 versus sham (7w); †*P* < 0.05 versus sham (9w)

SBP and heart rate immediately before sham surgery or RDN at early or advanced stages are shown in Table 1. For the combined sham and RDN groups, SBP was higher at 9 weeks than at 7 weeks but there was no significant difference in heart rate between 7 and 9 weeks. There was also no difference in SBP or heart rate between the sham and RDN groups at either 7 or 9 weeks. At 12 weeks of age, SBP was significantly lower in the RDN group than in the sham group for each stage of intervention (Fig. 1A, Table 2).

**Table 1:** Baseline hemodynamic measurements before sham surgery or RDN at 7 weeks (early stage) or 9 weeks (advanced stage) of age

Parameter	Early stage		Advanced stage	
	sham (7w) *n* = 8	RDN (7w) *n* = 11	sham (9w) *n* = 9	RDN (9w) *n* = 10
SBP (mmHg)	136.7 ± 3.3	135.6 ± 1.5	171.2 ± 2.0*	166.9 ± 3.0†
Heart rate (beats/min)	391.6 ± 5.9	405.3 ± 4.3	423.0 ± 10.4	408.9 ± 7.9

Data are means ± SEM for the indicated numbers of animals. *P < 0.05 versus sham (7w), †*P* < 0.05 versus RDN (7w)

**Table 2:** Physiological and echocardiographic parameters at 12 weeks of age for rats subjected to sham surgery or RDN at 7 weeks (early stage) or 9 weeks (advanced stage) of age

Parameter	Early stage		Advanced stage	
	sham (7w)	RDN (7w)	sham (9w)	RDN (9w)
Physiological parameters	*n* = 8	*n* = 11	*n* = 9	*n* = 10
Body weight (g)	328.7 ± 9.9	310.4 ± 8.1	325.5 ± 7.0	324.1 ± 6.1
SBP (mmHg)	194.5 ± 5.4	176.4 ± 2.8*	191.5 ± 4.5	179.8 ± 4.0†
Heart rate (beats/min)	416.6 ± 10.5	408.8 ± 5.2	422 ± 7.0	404.0 ± 10.0
Organ weights	*n* = 11	*n* = 11	*n* = 11	*n* = 12
Tibial length (TL, mm)	37.4 ± 0.3	37.5 ± 0.4	38.2 ± 0.3	37.8 ± 0.5
Heart weight/TL (mg/mm)	39.4 ± 1.2	37.2 ± 1.0	39.0 ± 1.0	38.0 ± 1.4
LV weight/TL (mg/mm)	29.5 ± 1.0	28.2 ± 0.8	29.7 ± 0.8	29.0 ± 1.1
Kidney weight/TL (mg/mm)	97.6 ± 2.5	93.3 ± 2.2	92.2 ± 2.9	96.0 ± 3.1
Retroperitoneal fat weight/TL (mg/mm)	66.7 ± 7.7	41.7 ± 5.0*	51.2 ± 4.7	64.3 ± 3.5†
Echocardiographic parameters	*n* = 7	*n* = 10	*n* = 9	*n* = 9
IVST (mm)	2.29 ± 0.11	2.43 ± 0.08	2.33 ± 0.06	2.32 ± 0.07
LVPWT (mm)	2.23 ± 0.09	2.23 ± 0.07	2.21 ± 0.06	2.19 ± 0.07
LVDd (mm)	7.35 ± 0.23	7.07 ± 0.18	7.29 ± 0.12	7.28 ± 0.14
LVDs (mm)	4.39 ± 0.41	4.09 ± 0.28	4.44 ± 0.15	4.36 ± 0.18
LVFS (%)	40.83 ± 4.39	42.58 ± 3.10	39.20 ± 1.69	40.29 ± 1.81
LVEF (%)	77.41 ± 3.74	79.60 ± 2.63	77.10 ± 1.87	78.14 ± 1.77
LV mass (mg)	933.07 ± 12.01	903.14 ± 9.89	921.20 ± 7.82	905.79 ± 11.67
RWT	0.62 ± 0.05	0.67 ± 0.04	0.63 ± 0.02	0.62 ± 0.03
*E*/*A*	1.26 ± 0.02	1.41 ± 0.05*	1.27 ± 0.04	1.43 ± 0.05†
IRT (ms)	48.40 ± 1.99	40.62 ± 1.25*	49.44 ± 2.00	42.76 ± 1.57†
Renal function parameters	*n* = 6	*n* = 4	*n* = 5	*n* = 4
Creatinine clearance (mL min^–1^ g^–1^ kidney weight)	0.67 ± 0.06	0.79 ± 0.10	0.74 ± 0.09	0.67 ± 0.02
Creatinine clearance (mL min^–1^ 100 g^–1^ body weight)	0.81 ± 0.06	0.90 ± 0.09	0.82 ± 0.10	0.79 ± 0.03
Sympathetic activity index	*n* = 6	*n* = 4	*n* = 5	*n* = 6
Urinary norepinephrine (μg/day)	1.29 ± 0.11	1.27 ± 0.15	1.62 ± 0.12	1.33 ± 0.20
Humoral immune parameter	*n* = 10	*n* = 7	*n* = 9	*n* = 10
Serum TNF-α (pg/mL)	19.65 ± 2.31	15.28 ± 2.98	15.55 ± 2.01	19.40 ± 2.09
Serum IL-6 (pg/mL)	153.6 ± 8.9	162.2 ± 10.3	168.4 ± 10.7	171.8 ± 12.1
Circulating renin-angiotensin system parameters	*n* = 4	*n* = 4	*n* = 4	*n* = 4
Plasma renin activity (ng/mL/hr)	4.18 ± 1.87	1.55 ± 0.10	3.78 ± 0.54	1.63 ± 0.16†
Plasma angiotensin II concentration (pg/mL)	10.74 ± 1.99	7.04 ± 1.23	13.22 ± 0.48	9.03 ± 0.47†

Data are means ± SEM for the indicated numbers of animals. *P < 0.05 versus sham (7w), †*P* < 0.05 versus sham (9w)

Renal norepinephrine content at 12 weeks was 66.73 ± 3.96 and 0.98 ± 0.30 ng/g tissue in rats subjected to sham surgery or RDN, respectively, at 7 weeks, whereas it was 68.06 ± 6.52 and 2.20 ± 1.04 ng/g tissue in those subjected to sham surgery or RDN, respectively, at 9 weeks (Fig. 1B). The percentage renal norepinephrine content at 12 weeks in rats subjected to RDN at 7 or 9 weeks relative to the mean value for corresponding sham-operated rats was thus 1.47 ± 0.45% (range 0.09–3.59%) and 3.23 ± 1.53% (range 0.17–12.17%), respectively. These results indicated that, with the exception of two rats (12.05% and 12.17%) subjected to RDN at 9 weeks, RDN was successful, on the basis that renal norepinephrine content in rats subjected to RDN was <10% of the mean value for sham-operated rats.

Body weight (Table 2), food intake (Fig. 1C), and heart rate (Table 2) at 12 weeks as well as the overall time courses of these parameters (data not shown) did not differ between the sham and RDN groups for either stage of intervention. Urinary Na^+^ excretion at 12 weeks was increased in the RDN (7w) group relative to the sham (7w) group, whereas it did not differ between the RDN (9w) and sham (9w) groups (Fig. 1D).

At 12 weeks of age, the ratio of heart or LV weight to tibial length (indices of cardiac and LV hypertrophy, respectively) did not differ between the sham and RDN groups for either stage (Table 2). The ratio of kidney weight to tibial length was also similar in the two groups for each stage. The ratio of visceral (retroperitoneal) fat weight to tibial length was smaller in the RDN group than in the sham group for the early stage, whereas it was higher in the RDN group than in the sham group for the advanced stage (Table 2).

### Echocardiographic data

Echocardiography revealed that the interventricular septum thickness (IVST), LV posterior wall thickness (LVPWT), LV end-diastolic and end-systolic diameters (LVDd and LVDs), LV fractional shortening (LVFS) and ejection fraction (LVEF), LV mass, and relative wall thickness (RWT) at 12 weeks of age were all similar in the sham and RDN groups for each stage (Table 2). The ratio of the peak flow velocity at the mitral level during rapid filling to that during atrial contraction (*E/A* ratio) was increased, whereas the isovolumic relaxation time (IRT) was decreased, in the RDN group compared with the sham group for both early and advanced stages. These data thus indicated that RDN at both stages preserved LV systolic function and ameliorated LV diastolic dysfunction, but did not affect LV hypertrophy or geometry, in salt-loaded DahlS rats.

### Cardiac pathology and gene expression

**Fig. 2:**
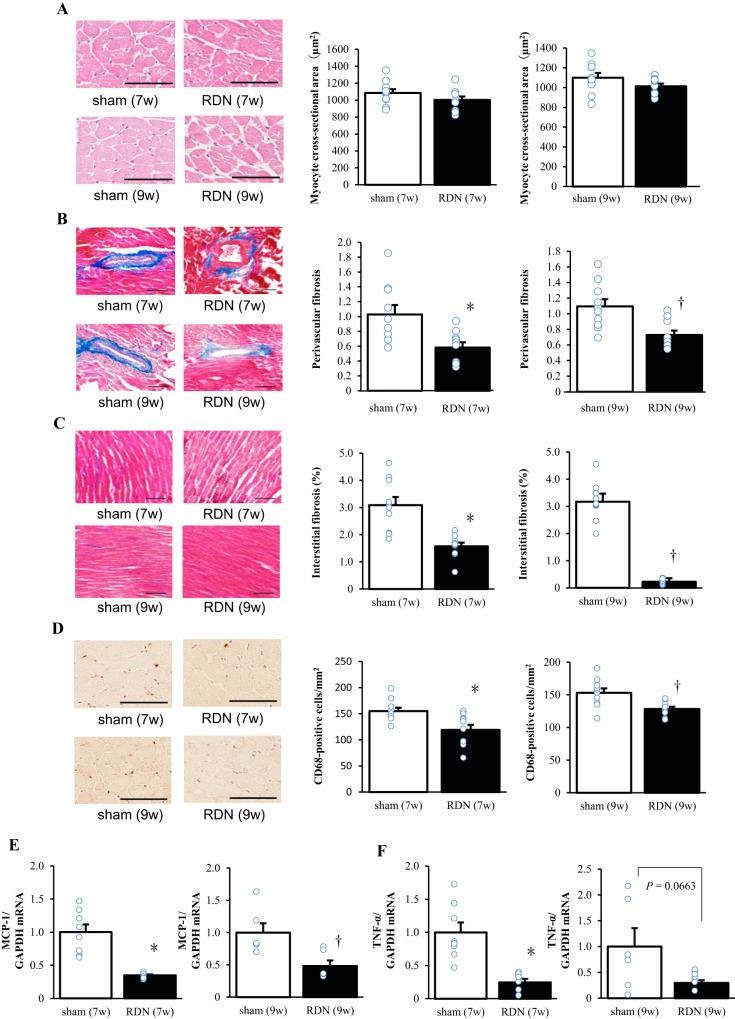
Cardiomyocyte size, cardiac fibrosis, macrophage infiltration, and inflammatory gene expression in the left ventricle at 12 weeks of age for rats subjected to sham surgery or RDN at 7 or 9 weeks of age. **A** Representative hematoxylin-eosin staining of transverse sections of the LV myocardium (scale bars, 50 µm) and cross-sectional area of cardiac myocytes determined from such sections. **B**, **C** Representative Azan-Mallory staining of collagen deposition in perivascular and interstitial regions of the LV myocardium (scale bars, 100 µm), respectively, as well as relative extents of perivascular and interstitial fibrosis determined from such images. **D** Representative immunohistochemical analysis of the monocyte-macrophage marker CD68 (scale bars, 100 µm) in the left ventricle and the density of CD68-positive cells as determined from such images. **E**, **F** Quantitative RT-PCR analysis of MCP-1 and TNF-α mRNAs, respectively, in the left ventricle. The amount of each mRNA was normalized by that of GAPDH mRNA and then expressed relative to the normalized value for the sham (7w) or sham (9w) group. All quantitative data are means + SEM [*n* = 10, 10, 10, and 10 (**A**‒**D**) or *n* = 8, 8, 6, and 6 (**E**, **F**) for sham (7w), RDN (7w), sham (9w), and RDN (9w) rats, respectively]. *P < 0.05 versus sham (7w); †*P* < 0.05 versus sham (9w)

Microscopic analysis revealed that the cross-sectional area of cardiac myocytes at 12 weeks of age did not differ between the sham and RDN groups for the early or advanced stage (Fig. 2A). In contrast, Azan-Mallory staining revealed that fibrosis in perivascular and interstitial regions of the LV myocardium was reduced in each RDN group compared with the corresponding sham group (Fig. 2B, C). Immunostaining for the monocyte-macrophage marker CD68 showed that macrophage infiltration in the LV myocardium was decreased in the RDN group compared with the sham group for both stages (Fig. 2D). Expression of the monocyte chemoattractant protein–1 (MCP-1) gene in the left ventricle was reduced in the RDN group compared with the sham group for both early and advanced stages (Fig. 2E), whereas that of the tumor necrosis factor–α (TNF-α) gene was significantly or tended to be (*P* = 0.0663) downregulated in the RDN group at the early or advanced stage, respectively (Fig. 2F).

### Renal function, pathology, and gene expression

**Fig. 3:**
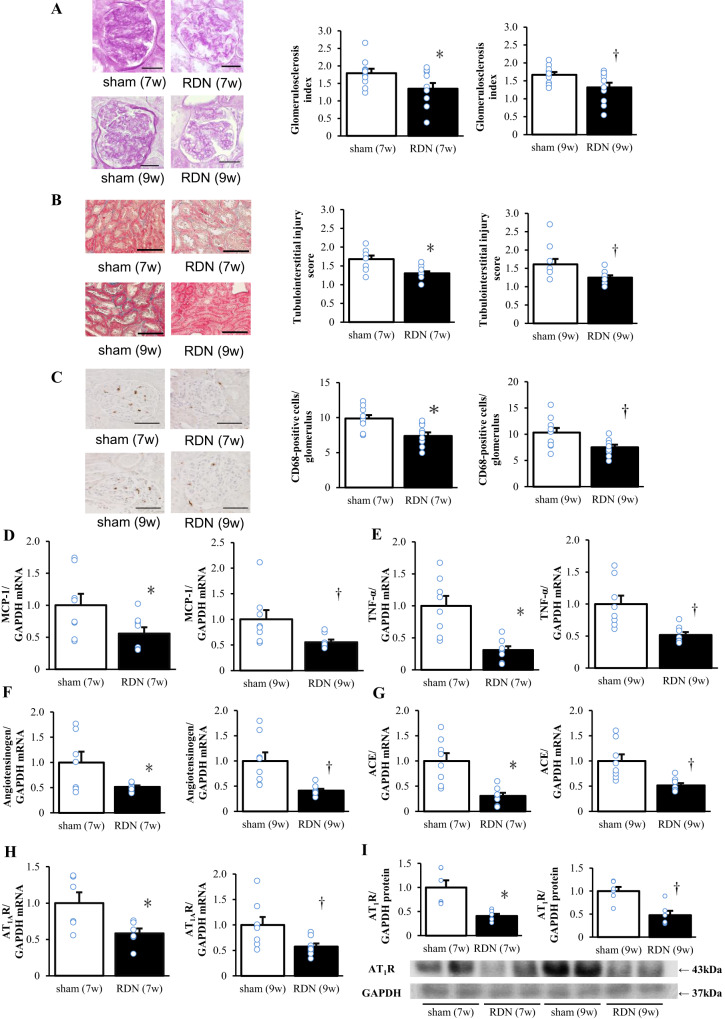
Histological changes and inflammatory and RAS-related gene expression in the kidney at 12 weeks of age for rats subjected to sham surgery or RDN at 7 or 9 weeks of age. **A** Representative periodic acid–Schiff staining of transverse sections of glomeruli (scale bars, 100 μm) and the glomerulosclerosis index (range of 0–4 reflects the extent of glomerulosclerosis) as determined from such sections. **B** Representative Azan-Mallory staining of transverse sections of tubulointerstitial regions (scale bars, 100 μm) and the tubulointerstitial injury score (range of 0–4 reflects the extent of tubulointerstitial injury) as determined from such sections. **C** Representative immunohistochemical analysis of the monocyte-macrophage marker CD68 in transverse sections of glomeruli (scale bars, 100 μm) and the number of CD68-positive cells in each glomerulus as determined from such sections. **D**–**H** Quantitative RT-PCR analysis of relative MCP-1 **D**, TNF-α **E**, angiotensinogen **F**, ACE **G**, and AT_1A_R **H** mRNA abundance in the kidney. **I** Representative immunoblot analysis of AT_1_R in the kidney as well as densitometric determination of the relative ratio of the amount of AT_1_R to that of GAPDH for such analysis. All quantitative data are means + SEM [*n* = 10, 10, 10, and 10 (**A**‒**C**), *n* = 8, 8, 8, and 8 (**D**, **E**, **F**), *n* = 7, 8, 8, and 8 (**F**), *n* = 6, 6, 8, and 8 (**H**), or *n* = 6, 6, 6, and 6 (**I**) for sham (7w), RDN (7w), sham (9w), and RDN (9w) rats, respectively]. *P < 0.05 versus sham (7w); †*P* < 0.05 versus sham (9w)

The ratio of creatinine clearance to either body or kidney weight at 12 weeks of age did not differ between the sham and RDN groups for early or advanced stages (Table 2). The glomerulosclerosis index, an indicator of the extent of glomerulosclerosis, as well as the tubulointerstitial injury score were lower in the RDN group than in the sham group for both stages (Fig. 3A, B). The number of CD68-positive cells in glomeruli (Fig. 3C) as well as the expression of MCP-1 (Fig. 3D) and TNF-α (Fig. 3E) genes in the kidney were all decreased in the RDN groups compared with the corresponding sham groups. In addition, with regard to the renin-angiotensin system (RAS), the amounts of angiotensinogen, angiotensin-converting enzyme (ACE), and angiotensin II type 1 A receptor (AT_1A_R) mRNAs (Fig. 3F–H) as well as that of angiotensin II type 1 receptor (AT_1_R) protein (Fig. 3I) were all downregulated in the RDN group for each stage.

### Serum and urine data

The serum concentrations of TNF-α and interleukin-6 (IL-6) in serum were similar in the sham and RDN groups for both early and advanced stages (Table 2). Urinary norepinephrine excretion also did not differ between the groups for either stage. Both renin activity and angiotensin II concentration in plasma were not significantly decreased in the RDN group for early stage (*P* = 0.21 for renin activity; *P* = 0.16 for angiotensin II concentration) (Table 2). In contrast, these parameters were significantly reduced in the RDN group for advanced stage.

### Adipose tissue pathology and gene expression

**Fig. 4:**
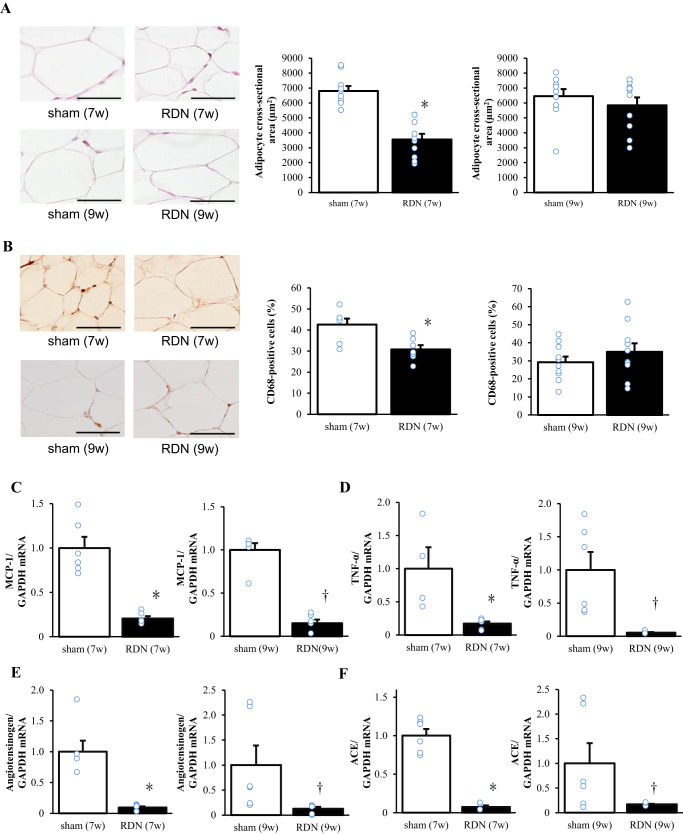
Histological changes and inflammatory and RAS-related gene expression in visceral (retroperitoneal) adipose tissue at 12 weeks of age for rats subjected to sham surgery or RDN at 7 or 9 weeks of age. **A** Representative hematoxylin-eosin staining of tissue sections (scale bars, 50 μm) and cross-sectional area of adipocytes determined from such sections. **B** Representative immunohistochemical staining of the monocyte-macrophage marker CD68 (scale bars, 100 µm) and the number of nuclei for CD68-positive cells as a percentage of total nuclei determined from such staining. **C**‒**F** Quantitative RT-PCR analysis of relative MCP-1 (**C**), TNF-α (**D**), angiotensinogen (**E**), and ACE (**F**) mRNA abundance. All quantitative data are means + SEM [*n* = 10, 10, 10, and 10 (**A**), *n* = 7, 7, 10, and 10 (**B**), *n* = 6, 6, 6, and 6 (**C, E**), *n* = 4, 6, 6, and 6 (**D**), or *n* = 6, 4, 6, and 6 (**F**) for sham (7w), RDN (7w), sham (9w), and RDN (9w) rats, respectively]. *P < 0.05 versus sham (7w); †*P* < 0.05 versus sham (9w)

Hematoxylin-eosin staining and immunostaining for CD68 revealed that adipocyte cross-sectional area and macrophage infiltration in visceral adipose tissue at 12 weeks of age were decreased specifically in the RDN (7w) group compared with the sham (7w) group (Fig. 4A, B). The expression of MCP-1 (Fig. 4C) and TNF-α (Fig. 4D) genes in visceral adipose tissue was decreased in the RDN group compared with the sham group for both stages, as were the amounts of angiotensinogen (Fig. 4E) and ACE (Fig. 4F) mRNAs.

## Discussion

We have here shown that RDN at both early and advanced stages of hypertension resulted in a moderate lowering of SBP as well as ameliorated LV inflammation, fibrosis, and diastolic dysfunction in salt-loaded DahlS rats. In addition, RDN at both stages attenuated renal injury and inflammation as well as downregulated RAS-related gene expression in the kidney. Furthermore, RDN at each stage inhibited the expression of both inflammatory cytokine and RAS-related genes in adipose tissue. The early intervention also reduced both fat mass and adipocyte size, whereas the late intervention increased fat mass without affecting adipocyte size. RDN at either stage did not affect circulating levels of inflammatory cytokines or urinary norepinephrine excretion. In contrast, RDN at advanced stage significantly reduced both renin activity and angiotensin II concentration in plasma.

Clinical studies have shown that RDN is safe and effective for lowering blood pressure in individuals with treatment-resistant hypertension [8, 9]. However, a subgroup analysis of the Symplicity HTN-3 trial [10] suggested that the anti-hypertensive effect of RDN was smaller in individuals with hypertension accompanied by chronic kidney disease (CKD) than in those without CKD. The DahlS rat is a model of hypertensive CKD and develops hypertension on a high-salt diet. However, DahlS rats fed a normal salt diet also manifest nephropathy [19], indicating that they develop renal injury even without high salt intake. In the present study, RDN at both the early and advanced stages of hypertension resulted in a moderate lowering of SBP in salt-loaded DahlS rats, whereas the intervention at either stage did not affect intake of the high-salt diet. Urinary Na^+^ excretion was increased by RDN at the early stage, but not by that at the advanced stage, with this difference possibly being related to the slightly greater anti-hypertensive effect of the intervention at the early stage. Pressure natriuresis is important in blood pressure control and is impaired in hypertension, and tubulointerstitial inflammation in the renal medulla can adversely affect pressure natriuresis directly or via increased renal sympathetic nerve activity, thereby giving rise to hypertension [20]. Renal medullary circulation plays a pivotal role in the development of salt-sensitive hypertension [21]. The influence of various regulatory factors on the control of arterial pressure is likely to be mediated partly through their impact on medullary blood flow, which is relatively insensitive to vasoconstrictors such as angiotensin II, endothelin-1 and norepinephrine, and renal sympathetic nerves [22]. Our results thus suggest that the anti-hypertensive effect of RDN at the early stage may be attributable, at least in part, to improved pressure natriuresis as well as amelioration of renal inflammation and injury in this model of hypertension with CKD. In contrast, RDN at the advanced stage also alleviated renal cortical damage but did not affect medullary injury or pressure natriuresis, resulting in an apparently smaller reduction in blood pressure. The restoration of baroreflex function by RDN at the advanced stage might have contributed to the moderate but significant lowering of blood pressure without improvement of pressure natriuresis [23].

A decrease in blood pressure can lead to a functional decline in estimated glomerular filtration rate as a result of a reduced perfusion pressure. However, RDN may improve renal perfusion by reducing intraparenchymal resistance, thereby having a potentially renal-protective effect [24]. Our findings that RDN at both stages induced a moderate lowering of SBP and ameliorated renal inflammation and injury, without affecting creatinine clearance, in salt-loaded DahlS rats are consistent with previous results [25, 26]. Our study thus suggests that a renal-protective effect of RDN may have offset a possible decline in creatinine clearance due to the modest reduction in blood pressure induced by the intervention.

The central nervous system regulates blood pressure. The rostral ventrolateral medulla (RVLM) contains neurons that play a key role in blood pressure regulation. RDN ablates both renal efferent sympathetic and afferent sensory nerves as well as reduces activity of the RVLM, leading to a reduction in blood pressure [27]. Increased sensitivity of the subfornical organ and the lamina terminalis to serum Na^+^ enhances the efferent sympathetic activity and blood pressure through activation of the paraventricular nucleus and the RVLM neurons, in essential hypertension. Also, the respiratory system modifies sympathetic activity in the hypothalamus and the nucleus tractus solitarius [28, 29]. The success of RDN performed at both stages in the present study was judged on the basis of a marked reduction in renal norepinephrine content [15]. Impaired renal Na^+^ excretion contributes to the pathogenesis of salt-sensitive hypertension [5]. Urinary norepinephrine excretion, an indicator of systemic sympathetic activity, was unaffected by RDN at either stage, consistent with a previous finding for SHR/NDmcr-cp (+/+) rats, a model of metabolic syndrome with hypertension [30]. The unchanged heart rate in animals subjected to RDN at either stage suggests that renal afferent nerves might not play a major role in systemic sympathetic activity in our model. However, we measured heart rate weekly by the tail-cuff method rather than continuous monitoring by telemetry [30]. Furthermore, the attenuation of cardiac and adipose tissue inflammation and injury by RDN at both stages, despite only a moderate reduction in blood pressure, is suggestive of a substantial role of renal afferent nerves in interactions between the kidney and multiple organ systems in salt-sensitive hypertension. However, we have no data on activities of the hypothalamus, RVLM neurons, and efferent systemic sympathetic nerves. Future studies are required to determine the possible role of central mechanisms in blood pressure regulation and organ cross talk in this model.

Recent observations from clinical trials have increased interest in interactions between the heart and kidney. Such organ cross talk is mediated by hemodynamic changes, oxidative stress, inflammation, and activation of the RAS and sympathetic nervous system [31]. The circulating concentrations of TNF-α and IL-6 and urinary norepinephrine excretion were not affected by RDN at either stage in our study, suggesting that these humoral inflammatory factors and systemic sympathetic activity did not substantially contribute to the reduction in organ inflammation induced by this intervention. In contrast, RDN at advanced stage reduced renin activity and angiotensin II concentration in plasma. In the heart and the kidney, the local RAS operates in close interaction with the circulating RAS [32]. Circulating renin may thus be taken up by tissues and most renin found in local RAS is derived from the kidney. Thus, although RDN moderately attenuated salt-induced hypertension and did not affect LV hypertrophy, its protective effect against renal injury related to inhibition of the kidney RAS might have contributed to amelioration of LV inflammation and injury, possibly through attenuation of systemic RAS.

RDN inhibited the expression of genes for proinflammatory cytokines and the RAS in the kidney and adipose tissue. Salt loading was previously shown to increase renal inflammation and fibrosis as well as to upregulate the expression of RAS-related genes in a model of CKD [6], in part through activation of a renocerebral RAS axis linked by renal afferent and efferent nerves [33]. In rats, CKD was found to be accompanied by activation of afferent sensory signals from the kidney and adipose tissue, with the local RAS and reactive oxygen species (ROS) in the brain also being linked with those in the kidney and adipose tissue through activation of efferent sympathetic nerves [34]. Salt loading in CKD enhanced the effects of these pathways by promoting the local RAS and ROS accumulation and consequent local inflammation in adipose tissue. Although we have no data on central mechanisms, it is possible that the anti-inflammatory effects of RDN on the kidney and adipose tissue are related to inhibition of the local RAS in the kidney and adipose tissue via the brain as well as systemic RAS.

Human and rodent adipose tissues contain all components of the RAS. Angiotensin II regulates adipocyte growth and differentiation, lipid metabolism, and the expression or release of adipokines and RAS components. Synthesis of angiotensinogen is a key step in the operation of the RAS in adipose tissue because it is released into the circulation [35]. RDN at the early stage of hypertension reduced both fat mass and adipocyte size in the present study, and these changes were accompanied by the downregulation of angiotensinogen and ACE gene expression, suggesting that RDN at this stage suppressed RAS-induced adipocyte growth. Indeed, in wild-type mice, targeted expression of angiotensinogen in adipose tissue increased fat mass, supporting the notion that adipocyte-derived angiotensinogen positively regulates adipose tissue growth [36].

In contrast, RDN at the advanced stage increased fat mass, without any change in adipocyte size, as well as attenuated the expression of these RAS-related genes. If renal sympathetic activity was higher at 3 weeks (at 9 weeks of age) than at 1 week (at 7 weeks of age) after initiation of the high-salt diet in DahlS rats, our finding that downregulation of angiotensinogen gene expression by RDN at the advanced stage was not accompanied by a reduction in adipocyte size would be consistent with the previous observation ^i^n a rat model of Parkinson’s disease that degeneration of dopaminergic neurons in the nigrostriatal system correlated with increased sympathetic activity and that such activity resulted in lipolysis and inhibition of fat cell differentiation [37]. β-Adrenergic–mediated lipolysis in adipocytes is associated with increased cellular levels of cyclic AMP, which increases angiotensinogen mRNA abundance in human adipose tissue [38]. The RAS may also contribute to the regulation of adipose differentiation, although conflicting findings have been obtained with regard to the possible effect of angiotensin II on adipocyte differentiation [38]. The differentiation of stem cells into adipocytes was associated with a decrease in angiotensinogen and ACE gene expression [39]. Although the reasons for the differential effects of RDN at the early and advanced stages on adipose pathology are unclear, it is possible that attenuation of the adipose RAS related to inhibition of circulating RAS by RDN at the advanced stage might have induced the differentiation of preadipocytes into small fat cells, thereby affecting macrophage polarization and downregulating inflammatory cytokine expression in adipose tissue [40].

Mediators whose levels are increased in the obese state, such as TNF-α and angiotensin II, have also been found to regulate angiotensinogen expression in adipocytes [41]. Dietary salt restriction reduced hypertension and circulating levels of inflammatory cytokines and insulin, as well as ameliorated adipose tissue inflammation and insulin signaling, without affecting fat mass, in obese DahlS rats [42], suggesting that these humoral factors are involved in the pathogenesis of salt-sensitive hypertension in these rats. However, circulating levels of TNF-α and IL-6 were not changed by RDN in our study, and the circulating concentration of angiotensin II was previously shown to be markedly reduced in salt-loaded DahlS rats at the stage of compensated LV hypertrophy [43]. Circulating inflammatory cytokines may therefore not play a major role in the adipose tissue response to RDN. Thus, attenuation of the circulating RAS by RDN could contribute to moderate blood pressure reduction as well as alleviation of LV, renal, and adipose tissue inflammation and injury through inhibition of the tissue RAS [44].

Although the effects of RDN on blood pressure lowering are inconsistent, it is consistently reported that RDN has not prevented the onset of hypertension in DahlS rats [7]. Indeed, combined surgical and chemical RDN before high salt intake reduced heart rate and attenuated the catabolic state, but it did not affect blood pressure elevation, in these rats [7]. On the other hand, chemical RDN after a high-salt diet attenuated cardiac remodeling and renal damage, but not hypertension, in DahlS rats [45]. Interestingly, although arterial pressure was lowered in Sprague-Dawley rats by RDN, it was increased to a similar extent in both denervated and intact animals by increased salt intake [46]. Although the reason for the discrepancy of the blood pressure lowering effect of RDN between the present and previous studies is unclear, it is possible that renal injury rather than renal sympathetic overactivity is related to blood pressure regulation in DahlS rats, as with obese DahlS rats [6]. Apart from differences in blood pressure measurement methods, the anti-hypertensive effect of RDN may also be affected by the time point and the procedure of RDN, as well as the concentration and the initiation time of a high-salt diet, in this model.

In conclusion, RDN moderately attenuated hypertension as well as ameliorated LV inflammation, fibrosis, and diastolic dysfunction in salt-loaded DahlS rats in a manner independent of the disease stage at which it was performed. RDN at both early and advanced stages also ameliorated renal inflammation and injury as well as downregulated RAS-related gene expression in the kidney. Furthermore, it reduced inflammatory cytokine and RAS-related gene expression in adipose tissue at both stages, but differentially affected adipose morphology in a stage-dependent manner. Although systemic inflammation and sympathetic activity were not affected by the intervention at either stage, the circulating RAS was suppressed by RDN at advanced stage. Our findings thus suggest the operation of cross talk among the kidneys, cardiovascular system, and adipose tissue possibly mediated via systemic RAS in salt-sensitive hypertension. Further studies are required to elucidate the mechanisms underlying the anti-hypertensive and organ-protective effects of RDN.

## Supplementary information

Supplementary Information

Supplementary Figure 1
