# Supplementary material for: Comparison of the effects of renal denervation at early or advanced stages of hypertension on cardiac, renal, and adipose tissue pathology in Dahl salt-sensitive rats
Source: Hypertens Res. 2024 Feb 15;47(10):2731–44. doi: 10.1038/s41440-024-01605-x (PMC11456506; doi:10.1038/s41440-024-01605-x)
Supplement: Supplementary file 1 — Supplementary Information [file 41440_2024_1605_MOESM1_ESM.docx]

**Supplementary Information**

Comparison of the effects of renal denervation at early or advanced stages of hypertension on cardiac, renal, and adipose tissue pathology in Dahl salt-sensitive rats

Kohzo Nagata, Kaito Tagami, Touko Okuzawa, Misaki Hayakawa, Akane Nomura, Tomo Nishimura, Katsuhide Ikeda, Kento Kitada, Shuhei Kobuchi, Yoshihide Fujisawa, Akira Nishiyama, Toyoaki Murohara

**Supplementary Methods**

**Histological and immunohistochemical analysis**

LV, kidney, or visceral (retroperitoneal) fat was analyzed by hematoxylin-eosin, Azan-Mallory, or periodic acid–Schiff (PAS) staining as well as by immunohistochemical staining for the monocyte-macrophage marker CD68 (antibody clone ED-1, diluted 1:100; Chemicon, Temecula, CA, USA), as previously described [1]. The glomerulosclerosis index was measured for 50 glomeruli in PAS-stained sections of each rat, also as described previously [1]. The tubulointerstitial injury score was evaluated in 10 fields of Azan-Mallory–stained sections for each rat [1]. CD68-positive cells were counted in 20 glomeruli for each rat, as previously described [1]. Image analysis was performed with NIH Scion Image software (Scion, Frederick, MD, USA) in a blinded manner relative to the experimental status of the animals [1].

**Biochemical analysis**

A blood sample was collected from the right carotid artery of rats that had been deprived of food overnight and then anesthetized by i.p. injection of pentobarbital sodium (40 mg/kg). The blood was centrifuged at 1400 × *g* for 10 min at room temperature, and the concentrations of TNF-α and interleukin-6 (IL-6) in serum were measured with the use of a rat TNF-α enzyme-linked immunosorbent assay kit (R&D Systems, Minneapolis, MN, USA) and a rat IL-6 enzyme-linked immunosorbent assay kit (R&D Systems, Minneapolis, MN, USA), respectively. Plasma renin activity was determined by enzyme immunoassay (Yamasa Corp., Chiba, Japan). Plasma angiotensin II concentration was determined by angiotensin II enzyme-linked immunosorbent assay kit (Enzo Life Sciences Inc., Farmingdale, NY, USA). At 12 weeks of age, rats were placed in a metabolic cage for the collection of 24-h urine specimens. The concentration of Na^+^ in urine was measured with a routine enzymatic assay. Urine volume and the ratio of creatinine clearance to kidney weight or body weight were determined by analysis of urine specimens. Creatinine clearance was calculated according to the standard formula *UV/P*, where *U* is the urinary creatinine concentration, *V* is the 24-h urine volume, and *P* is the serum creatinine concentration [2]. The concentration of norepinephrine in urine was measured by high-performance liquid chromatography, and urinary catecholamine excretion over 24 h was calculated as previously described [3].

**Quantitative RT-PCR analysis**

Total RNA was prepared from LV, renal, or visceral (retroperitoneal) fat tissue as described previously [4] and was subjected to RT with a PrimeScript RT Reagent Kit (Takara, Kusatsu, Japan). The resultant cDNA was amplified by real-time PCR analysis as performed with SYBR Mix Ex Taq II (Takara), a Thermal Cycler Dice Real Time System II (Takara), and specific primers (forward and reverse, respectively) for MCP-1 (5′- TATGCAGGTCTCTGTCACGC-3′ and 5′-GGCATTAACTGCATCTGGCTG-3′, GenBank accession no. [NM_031530.1](https://www.ncbi.nlm.nih.gov/entrez/viewer.fcgi?db=nucleotide&id=13928713)), TNF-α (5′-TTCTCATTCCTGCTCGTGGC-3′ and 5′-TTGAGAAGATGATCTGAGTGTGAGG-3′, GenBank accession no. NM_012675.3), angiotensinogen (5′-CCAGCACGACTTCCTGACTT-3′ and 5′-AGATTTGCCTCAGCACCCAA-3′, GenBank accession no. NM_134432.2), ACE (5′-CGTCCACCGTTACCAGACAA-3′ and 5′-TTGGCCTCTGCGTATTCGTT-3′, GenBank accession no. NM_012544.1), and AT_1A_R (5′-CTCTGCCACATTCCCTGAGTTA-3′ and 5′-ACTTTCTGGGAGGGTTGTGTG-3′, GenBank accession no. NM_030985.4). The abundance of mRNAs for target genes was normalized by the amount of glyceraldehyde-3-phosphate dehydrogenase (GAPDH) mRNA (NM_017008.4) [5].

**Immunoblot analysis**

Total protein was isolated from the kidney and quantitated [6], equal amounts of protein were subjected to SDS-polyacrylamide gel electrophoresis, and the separated proteins were transferred to a polyvinylidene difluoride membrane, as described previously [7]. The membrane was incubated first with a mouse monoclonal antibody to AT_1_R (1:500 dilution; sc-515884, Santa Cruz Biotechnology, Dallas, TX, USA) or a rabbit monoclonal antibody to GAPDH (1:1000; #2118, Cell Signaling Technology, Danvers, MA, USA), and then with horseradish peroxidase–conjugated horse antibodies to mouse immunoglobulin G (1:3000, #7076, Cell Signaling Technology) or goat antibodies to rabbit immunoglobulin G (1:1000, #7074, Cell Signaling Technology), respectively. Detection and quantification of immune complexes were performed as described [8].

**Supplementary References**

1. Aoyama K, Komatsu Y, Yoneda M, Nakano S, Ashikawa S, Kawai Y, et al. Alleviation of salt-induced exacerbation of cardiac, renal, and visceral fat pathology in rats with metabolic syndrome by surgical removal of subcutaneous fat. Nutr Diabetes. 2020;10:28.

2. Hattori T, Murase T, Ohtake M, Inoue T, Tsukamoto H, Takatsu M, et al. Characterization of a new animal model of metabolic syndrome: the DahlS.Z-Lepr(fa)/Lepr(fa) rat. Nutr Diabetes. 2011;1:e1.

3. Matsuura N, Nagasawa K, Minagawa Y, Ito S, Sano Y, Yamada Y, et al. Restraint stress exacerbates cardiac and adipose tissue pathology via beta-adrenergic signaling in rats with metabolic syndrome. Am J Physiol Heart Circ Physiol. 2015;308:H1275-86.

4. Matsuura N, Asano C, Nagasawa K, Ito S, Sano Y, Minagawa Y, et al. Effects of pioglitazone on cardiac and adipose tissue pathology in rats with metabolic syndrome. Int J Cardiol. 2015;179:360-9.

5. Ashikawa S, Komatsu Y, Kawai Y, Aoyama K, Nakano S, Cui X, et al. Pharmacological inhibition of the lipid phosphatase PTEN ameliorates heart damage and adipose tissue inflammation in stressed rats with metabolic syndrome. Physiol Rep. 2022;10:e15165.

6. Hattori T, Murase T, Takatsu M, Nagasawa K, Matsuura N, Watanabe S, et al. Dietary salt restriction improves cardiac and adipose tissue pathology independently of obesity in a rat model of metabolic syndrome. J Am Heart Assoc. 2014;3:e001312.

7. Xu J, Nagata K, Obata K, Ichihara S, Izawa H, Noda A, et al. Nicorandil promotes myocardial capillary and arteriolar growth in the failing heart of Dahl salt-sensitive hypertensive rats. Hypertension. 2005;46:719-24.

8. Komatsu Y, Aoyama K, Yoneda M, Ito S, Sano Y, Kawai Y, et al. Surgical ablation of whitened interscapular brown fat ameliorates cardiac pathology in salt-loaded metabolic syndrome rats. Ann N Y Acad Sci. 2021;1492:11-26.
