# Supplementary figures and images for: Comparison of the effects of renal denervation at early or advanced stages of hypertension on cardiac, renal, and adipose tissue pathology in Dahl salt-sensitive rats
Source: Hypertens Res. 2024 Feb 15;47(10):2731–44. doi: 10.1038/s41440-024-01605-x (PMC11456506; doi:10.1038/s41440-024-01605-x)

# Supplementary Figure 1

Full unedited gels for Figure 3I

AT<sub>1</sub>R

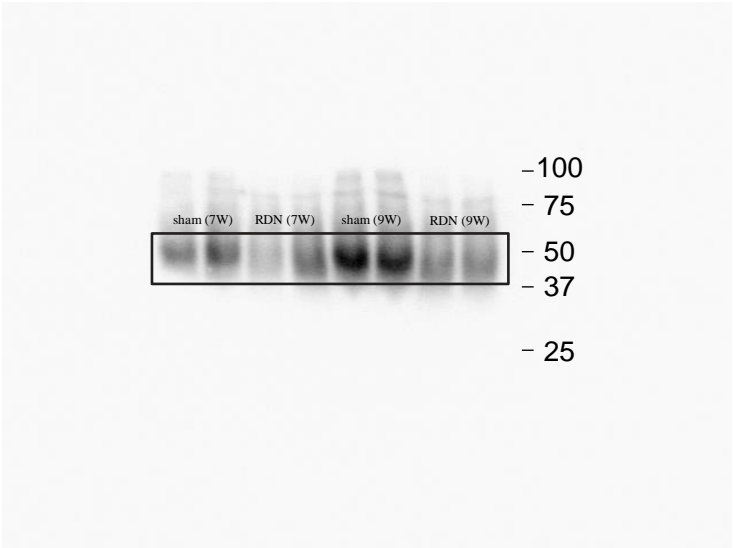

GAPDH

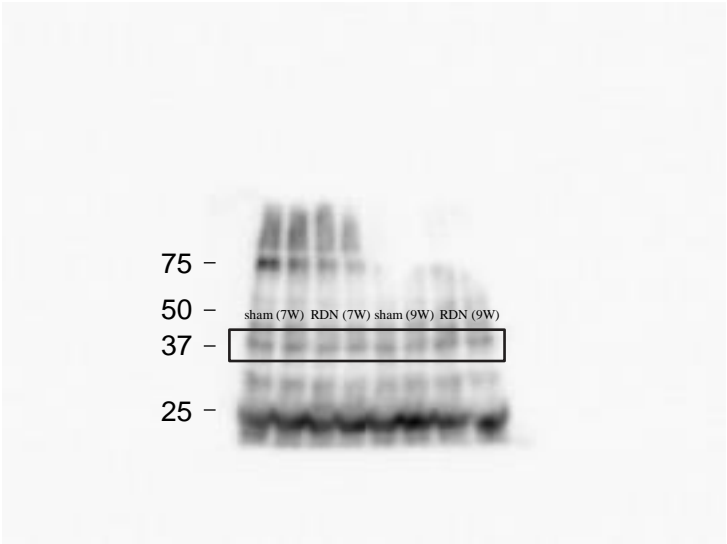

Supplement: Supplementary file 2 — Supplementary Figure 1 [file 41440_2024_1605_MOESM2_ESM.pdf]
